# Coumarins as Modulators of the Keap1/Nrf2/ARE Signaling Pathway

**DOI:** 10.1155/2020/1675957

**Published:** 2020-04-22

**Authors:** Emad H. M. Hassanein, Ahmed M. Sayed, Omnia E. Hussein, Ayman M. Mahmoud

**Affiliations:** ^1^Department of Pharmacology & Toxicology, Faculty of Pharmacy, Al-Azhar University-Assiut Branch, Egypt; ^2^Biochemistry Laboratory, Chemistry Department, Faculty of Science, Assiut University, Egypt; ^3^Physiology Division, Zoology Department, Faculty of Science, Beni-Suef University, Egypt

## Abstract

The Keap1/Nrf2/ARE system is a central defensive mechanism against oxidative stress which plays a key role in the pathogenesis and progression of many diseases. Nrf2 is a redox-sensitive transcription factor controlling a variety of downstream antioxidant and cytodefensive genes. Nrf2 has a powerful anti-inflammatory activity mediated via modulating NF-*κ*B. Therefore, pharmacological activation of Nrf2 is a promising therapeutic strategy for the treatment/prevention of several diseases that are underlined by both oxidative stress and inflammation. Coumarins are natural products with promising pharmacological activities, including antioxidant, anticancer, antimicrobial, and anti-inflammatory efficacies. Coumarins are found in many plants, fungi, and bacteria and have been widely used as complementary and alternative medicines. Some coumarins have shown an ability to activate Nrf2 signaling in different cells and animal models. The present review compiles the research findings of seventeen coumarin derivatives of plant origin (imperatorin, visnagin, urolithin B, urolithin A, scopoletin, esculin, esculetin, umbelliferone, fraxetin, fraxin, daphnetin, anomalin, wedelolactone, glycycoumarin, osthole, hydrangenol, and isoimperatorin) as antioxidant and anti-inflammatory agents, emphasizing the role of Nrf2 activation in their pharmacological activities. Additionally, molecular docking simulations were utilized to investigate the potential binding mode of these coumarins with Keap1 as a strategy to disrupt Keap1/Nrf2 protein-protein interaction and activate Nrf2 signaling.

## 1. Introduction

Coumarins are organic compounds in the benzopyrone class. This group comprises a large number of compounds that are widely distributed in the plant kingdom and have the highest concentration in fruits, seeds, roots, and leaves [1]. The name coumarin has been derived from the French word “Coumarou,” the common name of tonka beans (*Dipteryx odorata*) [2, 3]. The number of coumarins identified as secondary metabolites in bacteria, fungi, and about 150 species of plants has been estimated to be more than 1300 compounds [4]. Coumarins are most abundant in those plants taxonomically assigned to the *Apiaceae*, *Asteraceae*, and *Rutaceae* families and play a significant role in human health. Coumarins have been widely used in complementary and alternative medicine and possess a diversity of pharmacological activities with low cost and few side effects [5–7]. Carrot, cherries, citrus fruits, apricots, celery, parsnip, and strawberries, as well as spices like cinnamon and fennels are some of the commonly consumed coumarin-containing phytofoods [8].

Natural coumarins are classified into 6 main types based on their chemical structure. These include simple coumarins, furanocoumarins, dihydrofuranocoumarins, phenylcoumarins, pyranocoumarins, and biscoumarins [6]. All have a coumarin core and are characterized by structural diversity which could be considered for drug discovery and development of therapeutic agents for multiple diseases [9–12]. In plants, coumarins have been suggested to function as growth regulators and bacterio- and fungistatic agents [13]. In addition, coumarins possess a broad range of pharmacological activities basically relying on the type of coumarin nucleus. The beneficial effects of coumarins include antimicrobial [14–17], antimutagenic [12, 18], anti-inflammatory [19, 20], anticoagulant [21], antithrombotic [22–24], vasodilatory [25, 26], and anticancer activities [27]. Inhibition of matrix metalloproteinases (MMPs) and cancer cell growth, migration, and invasion and induction of apoptosis have been demonstrated as the effects underlying the anticancer activity of coumarins [28, 29]. Coumarins have also shown antihyperglycemic, antifibrotic, antiadipogenic, and cytochrome P450 inhibitory activities [30–34]. In a mouse model of cerebral ischemia/reperfusion (I/R) injury, the coumarin esculetin showed a potent neuroprotective effect when administered intracerebroventricularly [35].

The antioxidant and anti-inflammatory activities of coumarins have been well-acknowledged in several *in vitro* and *in vivo* studies [36, 37]. Coumarins suppress oxidative stress through their ability to scavenge reactive oxygen species (ROS) and inhibit neutrophil-dependent superoxide anion generation and lipid peroxidation. Moreover, coumarins can effectively reduce tissue edema-associated inflammation through suppressing both lipoxygenase and cyclooxygenase enzymatic activities and prostaglandin synthesis and release [20, 38–40].

**Figure 1:**
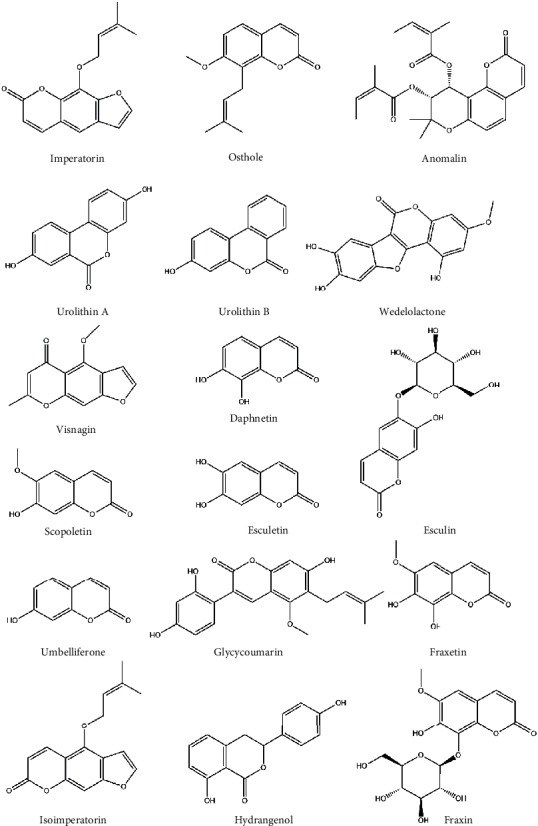
Chemical structure of selected coumarin-derived compounds.

Oxidative stress is a state of imbalance between the production of free radicals and their degradation by antioxidants. This redox imbalance occurs as a result of increased ROS generation and diminished antioxidant defenses. Although produced normally through different metabolic processes, excess ROS can provoke inflammation and damage lipids, proteins, and other cellular macromolecules, leading to oxidative stress and cell death. Therefore, oxidative stress is implicated in the pathogenesis of a wide range of metabolic disorders and chronic diseases [41–43]. Given their ability to suppress excessive ROS generation and enhance antioxidants [30, 34, 44], the pharmacologic effects of coumarins could be mediated through their antioxidant efficacy. The present review presents an overview of the modulatory role of a number of plant-derived coumarins (Figure 1) on nuclear factor (erythroid-derived 2)-like 2 (Nrf2), a transcription factor which protects against oxidative injury and inflammation [45]. In addition, we investigated the potential binding mode of coumarins to Kelch-like ECH-associated protein 1 (Keap1) as a strategy to disrupt Keap1/Nrf2 protein-protein interaction (PPI) using molecular docking simulations.

## 2. Keap1/Nrf2/ARE Signaling Pathway

**Figure 2:**
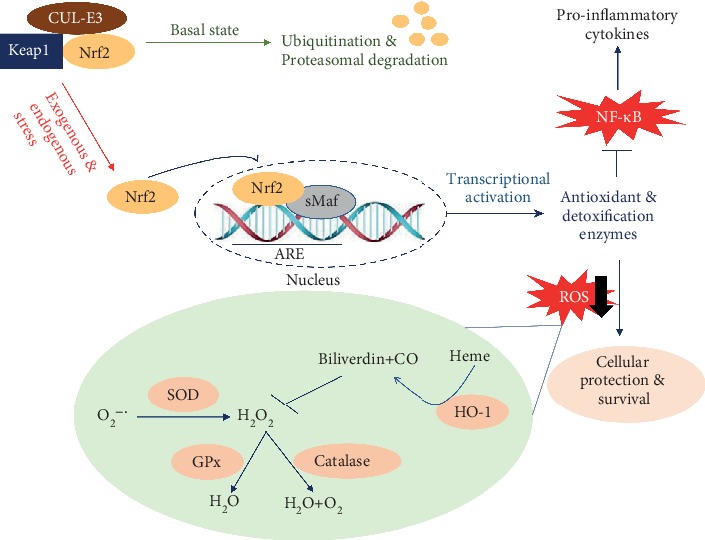
Nrf2 activity is augmented by exogenous and/or endogenous stressors. Under basal conditions, Keap1 mediates Nrf2 ubiquitination and subsequent proteasomal degradation through acting as an adaptor molecule for CUL-E3 ligase. Upon exposure to exogenous and/or endogenous stressors, such as xenobiotics and ROS, respectively, Nrf2 translocates into the nucleus and binds to the ARE to activate cytoprotective molecules, including antioxidant and detoxification enzymes. Superoxide dismutase (SOD) mediates the dismutation of superoxide radicals (O_2_^-·^) leading to the formation of hydrogen peroxide (H_2_O_2_). Catalase and glutathione peroxidase (GPx) catalyze the degradation of H_2_O_2_. HO-1 catalyzes degradation of heme to biliverdin and bilirubin which are potential antioxidants [49]. CO: carbon monoxide.

Nrf2 is a transcription factor generally known to enhance the cellular defense system to counteract oxidative injury and inflammation. In conditions without oxidative stimuli, Nrf2 exists in the cytoplasm sequestered by Keap1 [46]. Keap1 mediates Nrf2 ubiquitination and subsequent proteasomal degradation through acting as an adaptor molecule for the CUL-E3 ligase. The dissociation of Keap1 from the CUL-E3 ligase is elicited upon exposure to electrophilic/oxidative stress which modifies the cysteine residues of Keap1, in particular Cys151, leading to accumulation of Nrf2 [47]. As a result, Nrf2 liberates and translocates into the nucleus where it binds to the antioxidant response element (ARE) and promotes the transcription of antioxidant genes [48] (Figure 2). Nrf2-target antioxidant genes include heme oxygenase-1 (HO-1), NAD(P)H dehydrogenase quinone 1 (NQO1), *γ*-glutamyl cysteine ligase modulatory and catalytic subunits (GCLM and GCLC, respectively), and ferritin, which function to maintain the oxidant/antioxidant balance inside the cells [48]. Thus, activation of Nrf2 is an effective strategy to suppress oxidative stress.

Besides attenuation of oxidative stress, Nrf2 activation can effectively prevent inflammation. Nrf2 signaling is regarded as the most sensitive redox pathway linked to oxidative injury and nuclear factor-*κ*B (NF-*κ*B), a master regulator of proinflammatory mediators. Both are redox-sensitive factors where NF-*κ*B is activated in oxidative stress conditions, and the lack of Nrf2 resulted in increased oxidative/nitrosative stress and subsequently amplification of cytokine production [50]. The crosstalk between Nrf2 and NF-*κ*B has been reviewed by Wardyn et al. [51]. The lack of Nrf2 can aggravate NF-*κ*B activity leading to increased inflammatory cytokine release [52], whereas Nrf2 upregulation resulted in diminished inflammatory responses in rodent models of liver and kidney injury [53–60]. The Nrf2 target gene HO-1 has been demonstrated to inhibit NF-*κ*B-mediated transcription of adhesion molecules possibly through decreasing free intracellular iron in endothelial cells [61]. NF-*κ*B activity can regulate Nrf2-mediated antioxidant gene expression. In this context, Yu et al. [62] have demonstrated that overexpression of the canonical NF-*κ*B subunit p65 increased nuclear Keap1 levels resulting in decreased Nrf2/ARE signaling. Given that Keap1 is mostly localized in the cytosol and proteins larger than 40 kDa cannot enter through the nuclear envelope, the nuclear translocation of Keap1 has been suggested to occur via interaction with karyopherin alpha 6 (KPNA6). In this context, overexpression of KPNA6 has been associated with decreased HO-1 and NQO1 [63]. Although different mechanisms of the inhibitory effect of NF-*κ*B p65 on Nrf2 have been postulated, a competition for the transcriptional coactivator CBP-p300 complex is the best supported mechanism. CBP-p300 has an intrinsic acetyl transferase activity and acetylates Nrf2 and p65 [64, 65].

## 3. The Modulatory Effect of Coumarins on Nrf2

In this section, we reviewed studies showing the role of Nrf2 signaling in mediating the pharmacologic effects of coumarins. Only coumarin derivatives reported to modulate Nrf2 signaling were included.

### 3.1. Imperatorin (IMP)

IMP (9-(3-methylbut-2-enoxy)furo[3,2-g]chromen-7-one) is a furanocoumarin present in high concentration in plants of the genus *Angelica* such as *Angelica archangelica* and *Angelica dahurica*. The pharmacological properties of IMP make it a promising candidate for drug development. It has been documented to possess antioxidant [66, 67], anti-inflammatory [68, 69], anticancer [68, 70], antibacterial [71], and antiallergic activities [72]. Singh et al. reported that IMP treatment significantly reduced the nociceptive behavior provoked with formalin and acetic acid [73]. IMP effectively reduced the lipopolysaccharide- (LPS-) induced rise in IL-1*β* and TNF-*α* levels in mice in a dose-dependent manner [73]. In a mouse model of paw edema induced by carrageenan, a potent anti-inflammatory activity of IMP was evident 4 h after the injection of carrageenan [73]. In ovalbumin- (OA-) challenged mice and LPS-induced dendritic cells (DCs), the antiallergic and anti-inflammatory effects of IMP were demonstrated [72]. In asthmatic mice, IMP, in a dose-dependent manner, reduced IgE levels, airway hyperresponsiveness, and Th2 cytokines and increased IL-10-producing T cells. In LPS-stimulated DCs, IMP increased IL-10 and suppressed the release of proinflammatory cytokines [72]. Li et al. have reported that IMP effectively diminished COX-2, IL-6, TNF-*α*, and iNOS expression via downregulation of NF-*κ*B and JAK/STAT signaling in alveolar macrophages [74]. In addition, IMP relieved zymosan-induced immune cell infiltration in mice, thereby attenuating lung inflammation, edema, and fibrosis [74]. Although these studies have documented the potent anti-inflammatory activity of IMP, none of them have pointed to the involvement of Nrf2 signaling. The ability of IMP to activate Nrf2 signaling has been evaluated in few studies. By using HepG2 stably transfected with ARE reporter and Nrf2 knockout mice, Prince et al. have studied whether IMP activates hepatic Nrf2. The results showed that IMP increased hepatic GST activity via the Nrf2/ARE mechanism [75]. Hu et al. reported that IMP-induced Nrf2 activation effectively upregulated its downstream antioxidants, possibly offering cellular protection to the heart from injury induced by arsenic trioxide (As_2_O_3_) [76]. Very recently, Xian et al. investigated the protective effect of IMP against excess ROS and chronic airway inflammation in OA-administered mice [77]. Treatment with IMP suppressed ROS, proinflammatory cytokines, inflammatory cell infiltration, collagen deposition, and goblet cell hyperplasia. These effects have been suggested to be mediated via modulation of Nrf2 signaling following IMP administration [77].

### 3.2. Visnagin

Visnagin (4-methoxy-7-methylfuro[3,2-g]chromen-5-one) is a bioactive compound extracted from *Ammi visnaga* fruits [78]. Visnagin possesses widespread pharmacological activities, including hypotensive and smooth muscle relaxation. In a dose-dependent manner, visnagin decreased blood pressure when administered intravenously. In isolated mesenteric arteries precontracted with noradrenaline, visnagin treatment resulted in a concentration-dependent relaxation [79]. In isolated rat aortic rings, visnagin inhibited the vascular smooth muscle contraction induced by different agents [80]. Visnagin has also shown protective effects against doxorubicin cardiotoxicity mediated via cytochrome P450 family 1 (CYP1) inhibition [81] and modulation of mitochondrial malate dehydrogenase [82]. In addition, visnagin prevented the deposition of renal crystals in hyperoxaluric rats [83]. Besides these activities, the antioxidant and anti-inflammatory activities of visnagin have been demonstrated. In this context, Lee et al. have examined the anti-inflammatory activity of visnagin in BV2 microglial cells challenged with LPS. The obtained results demonstrated that visnagin ameliorates LPS-induced TNF-*α*, IL-1*β*, iNOS, IFN-*γ*, and IL-6 expression via a NF-*κ*B-dependent mechanism and increases the production of the anti-inflammatory cytokine IL-10 [84]. A recent study by Khalil et al. evaluated the anti-inflammatory activity of visnagin using molecular docking simulation and *in vivo* and *in vitro* assays [85]. Visnagin exhibited a strong anti-inflammatory activity with a high safety profile *in vivo* and showed an *in vitro* selective COX-2 inhibition [85]. The role of Nrf2 in mediating the anti-inflammatory and antioxidant activities of visnagin has been recently reported by Pasari et al. using a model of cerulein-induced acute pancreatitis in mice [86]. Visnagin decreased the expression of IL-1*β*, TNF-*α*, IL-6, and IL-17 in a dose-dependent manner. Importantly, visnagin enhanced the antioxidant defenses by effective upregulation of Nrf2 and mitigated pancreatic inflammation through suppression of the NF-*κ*B expression in the acinar cells. Additionally, visnagin inhibited the release of inflammatory cytokines in pulmonary and intestinal tissues [86].

### 3.3. Urolithin B

Urolithin B (3-hydroxybenzo[c]chromen-6-one) is one of the gut microbial metabolites of ellagitannins, a class of compounds found in many plants, including medicinal herbs, pomegranates, and tropical fruits [87, 88]. Ellagitannin is hydrolyzed to ellagic acid which is then catabolized by microflora in the intestinal tract into urolithin derivatives (urolithin A-D) [89]. Urolithin B is the final product catabolized among the urolithin derivatives that possesses anticancer activity on prostate [90] and colon cancer [91]. Treatment with urolithin B induced apoptosis of HT-29 colon cancer cells *in vitro* [91]. The anticancer activity of urolithin B was mediated through disruption of the mitochondrial membrane potential and activation of caspases 8, 9, and 3 [91]. In addition, urolithin B exhibited potent anti-inflammatory [92] and antioxidant effects [93] and has been reported to attenuate neurotoxicity in Alzheimer's disease by preventing *β*-amyloid fibrillation *in vitro* [94]. The dual anti-inflammatory and antioxidant effect of urolithin B has been recently investigated by Lee et al. [92] in microglia challenged with LPS. Urolithin B reduced cytokine release and NO production, while it significantly increased the expression of IL-10. In addition, urolithin B significantly decreased TNF-*α*, IL-6, and NO production in stimulated BV2 cells. Additionally, the *in vivo* studies reported that urolithin B inhibited LPS-induced microglia activation in the mouse brain [92]. The study has also scrutinized the antioxidant activity of urolithin B. The results showed a strong antioxidant activity mediated by reducing intracellular ROS production and NADPH oxidase subunit expression through upregulation of Nrf2/ARE signaling and HO-1 expression. Interestingly, urolithin B suppressed the activity of NF-*κ*B by reducing I*κ*B*α* phosphorylation. In addition, urolithin B inhibited both ERK and JNK phosphorylation while it enhanced the AMPK phosphorylation which is associated with a dual anti-inflammatory and antioxidant activity [92].

### 3.4. Urolithin A

Urolithin A (3,8-dihydroxybenzo[c]chromen-6-one) is the one of the main microbiota metabolites of pomegranate ellagitannins. It is characterized by high safety as evidenced by a previous study carried out by Heilman et al. [95]. Urolithin A has a wide range of bioactivities, such as antioxidant [96], anti-inflammatory [97, 98], and anticancer effects [99]. In human colonic fibroblasts stimulated with IL-1*β*, urolithin A inhibited PGE2 production, downregulated COX-2, and suppressed NF-*κ*B nuclear translocation [97]. In human aortic endothelial cells exposed to TNF-*α*, urolithin A showed a great ability to inhibit monocyte adhesion, chemokine expression, and cell migration [98]. Urolithin A improved the gut microbiota in high fat diet- (HFD-) fed rats and reversed the LPS-induced inflammatory response in Caco2 cells [100]. Fu et al. have conducted a study to examine the role of urolithin A on IL-1*β*-induced inflammatory response in human osteoarthritis through *in vitro* and *in vivo* approaches. In human chondrocytes *in vitro*, urolithin A prevented the IL-1*β*-induced overproduction of TNF-*α*, IL-6, PGE2, COX-2, iNOS, and NO in a concentration-dependent manner via suppressing NF-*κ*B activation [101]. Another recent study carried out by Gong et al. concluded that urolithin A attenuates memory impairment and neuroinflammation in APP/PS1 mice via suppressing p38 MAPK and NF-*κ*B p65 activation along with enhancing cerebral AMPK activation [102]. Two recent studies have reported the impact of Nrf2 activation by urolithin A. The first study was done by Liu et al. who demonstrated that urolithin A is a promising antiaging agent through potent inhibition of intracellular ROS promoted by activation of the Nrf2-mediated antioxidative response [103]. The second study done by Singh et al. concluded that urolithin A can be used for the treatment of colitis through remedying barrier dysfunction [104]. This study showed the anti-inflammatory effects of urolithin A and highlighted the role of the activation of Nrf2-dependent pathways [104].

### 3.5. Scopoletin

Scopoletin (7-hydroxy-6-methoxychromen-2-one) is a natural coumarin found in many medicinal plants, including members of the genus *Scopolia* as well as species of the *Artemisia*, *Brunfelsia*, *Solanum*, and *Mallotus* and other genera. Scopoletin is present in many edible plants and foods, such as oats, garlic, lemon, grapefruit, celery, red pepper, chili pepper, carrots, chicory, and bael [105–107]. It possesses many pharmacological functions, including antimicrobial [108], antiaging [109], anti-inflammatory [110, 111], and antioxidant effects [112, 113]. Scopoletin is also known for its cytotoxic activity against different cancer cells [114, 115].

Scopoletin has also shown antihyperglycemic activity in diabetic mice and inhibited *α*-glucosidase *in vitro* [116]. The antidiabetic activity of scopoletin has been suggested to be exerted via inhibition of the carbohydrate digestive enzymes [116]. In the same context, scopoletin improved insulin sensitivity and enhanced glucose uptake through activation of GLUT4 translocation and PI3K and AMPK signaling in 3T3-L1 adipocytes *in vitro* [117]. The supplementation of scopoletin prevented hepatic steatosis in diabetic mice by inhibiting lipid biosynthesis and TLR4-MyD88 pathways [118]. The anti-inflammatory property of scopoletin was shown *in vivo* in various animal studies [110, 111, 118, 119]. The anti-inflammatory activity of scopoletin has been reported to involve suppression of myeloperoxidase (MPO), a neutrophil infiltration biomarker; adenosine-deaminase (ADA); TNF-*α*; IL-1*β*; and NO via inhibition of the NF-*κ*B and p38 MAPK phosphorylation. In a rat model of osteoarthritis, scopoletin downregulated collagenases and reduced the proinflammatory mediators in a dose-dependent manner [119]. In human fibroblasts, scopoletin inhibited p38 phosphorylation, MMP-1, NF-*κ*B, MAPK, and the mRNA abundance of IL-1*α* and TNF-*α* [120]. In a mouse model of cerulein-induced acute pancreatitis and lung injury, scopoletin suppressed pancreatic and pulmonary TNF-*α*, IL-1*β*, mast cell activation, and NF-*κ*B signaling [111]. The ability of scopoletin to upregulate Nrf2/HO-1 signaling has been supported by few studies. Given the antidiabetic efficacy of scopoletin, Chang et al. have investigated its insulin sensitizing and antiglycation effects in diabetic rats, pointing to the role of Nrf2 signaling [121]. Scopoletin suppressed the formation of advanced glycation end products (AGEs), hyperglycemia, and insulin resistance and enhanced Nrf2, Akt, and GLUT2 in hepatocytes [121]. Very recently, Narasimhan et al. reported that scopoletin protected against oxidative stress and apoptosis induced by rotenone via Nrf2 activation and investigated its neuroprotective effects for Parkinson's disease in a rat model and *in vitro* using SH-SY5Y cells [122].

### 3.6. Daphnetin

Daphnetin (7,8-dihydroxychromen-2-one) is one of the coumarin derivatives extracted from *Daphne Korean Nakai* [123, 124]. It has been clinically used in the treatment of rheumatoid arthritis, lumbago, and coagulation disorders and as an antipyretic [125–127]. It possesses multiple pharmacological properties, including anti-inflammatory and oxidant activities [128, 129], and demonstrated a significant anticancer effect *in vitro* [130, 131]. In a rat model of collagen-induced arthritis, daphnetin suppressed joint destruction, synovial hyperplasia, and Th1/Th2/Th17-type cytokines in splenic lymphocytes and increased the expression of Foxp3 [125], demonstrating a potent anti-inflammatory activity. Treatment of arthritic rats with daphnetin resulted in reduced levels of TNF-*α*, IL-1*β*, and macrophage migration inhibitory factor (MIF) [126]. Shen et al. reported that daphnetin decreased LPS-induced inflammation and reduced endotoxin lethality in mice. In this study, daphnetin suppressed TNF-*α*, IL-1*β*, IL-6, NO, and PGE2 release along with the expression of iNOS and COX-2 and inhibited ROS production in Raw264.7 cells [129].

Zhang et al. have demonstrated the protective effect of daphnetin against cisplatin nephrotoxicity via suppressing oxidative injury and inflammation [132]. Daphnetin significantly inhibited cisplatin-induced ROS generation, lipid peroxidation, NF-*κ*B activation, and the levels of IL-1*β* and TNF-*α* production in a dose-dependent manner. These effects have been accompanied with upregulation of Nrf2 and HO-1 expression [132]. Another study by Liu et al. reported that daphnetin protected against NAFLD *in vitro* in oleic acid-treated hepatocytes through Nrf2 activation while it effectively decreased CYP2E1 and CYP4A expression [133]. Daphnetin showed a potent inhibitory effect on oleic acid-induced ROS generation and promoted glucose uptake, insulin sensitivity, and PI3K/Akt signaling in hepatocytes [133]. In support of these findings, daphnetin mitigated oxidative stress in human lung epithelial cells exposed to arsenic through Keap1 protein downregulation and marked activation of the Nrf2-dependent antioxidant response with a dramatic upregulation of the ARE in a dose-dependent manner [134]. Daphnetin inhibited oxidative stress and inflammatory response in high glucose- (HG-) stimulated human glomerular mesangial cells (MCs) [135]. Daphnetin strikingly reduced ROS and decreased the production of IL-1*β*, IL-6, and TNF-*α* via suppression of the NF-*κ*B pathway. Mechanistically, daphnetin positively upregulated Nrf2 while it inhibited the expression Keap1 in HG-stimulated MCs [135]. Zhi et al. reported that daphnetin protected hippocampal neurons exposed to oxygen-glucose deprivation-induced injury against I/R via marked enhancement of the nuclear translocation of Nrf2 and HO-1 expression [136]. Moreover, Nrf2 knockdown blocked the protective effect of daphnetin on I/R in hippocampal neurons, confirming the critical role of Nrf2/HO-1 signaling activation in the neuroprotective effect of daphnetin [136]. Lv and coworkers demonstrated that daphnetin effectively inhibited cytochrome c release and NLRP3 inflammasome activation through upregulation of the Nrf2 nuclear translocation along with Keap1 protein downregulation [137]. Additionally, daphnetin suppressed ROS generation induced by *tert*-butyl hydroperoxide (t-BHP) which is mostly blocked in Nrf2 knockout macrophages. Accordingly, daphnetin has a protective role against t-BHP-induced oxidative injury via the Nrf2/ARE signaling pathway [137]. Furthermore, daphnetin ameliorated carbon tetrachloride- (CCl_4_-) induced hepatotoxicity in rats through induction of the nuclear translocation of Nrf2, thus inducing HO-1 expression [138]. These studies supported the notion that Nrf2 activation is critical for the protective mechanism of daphnetin against oxidative injury and inflammation induced by several insults.

### 3.7. Esculin

Esculin (7-hydroxy-6-[(2*S*,3*R*,4*S*,5*S*,6*R*)-3,4,5-trihydroxy-6-(hydroxymethyl)oxan-2-yl]oxychromen-2-one) is a coumarin derivative found in *Aesculus hippocastanum L.* (horse-chestnut). The anti-inflammatory [139] and antioxidant [140, 141] activities of esculin have been well-acknowledged. By using the *in vitro* ABTS, ORAC, and DPPH assays, Zhang et al. have demonstrated the potent antiradical activity of esculin [141]. In a rat model of colon carcinogenesis, esculin mitigated oxidative stress, DNA damage, and tumorigenesis [140]. Additionally, esculin showed wide pharmacological activities against different diseases, such as cognitive impairment in experimental diabetic nephropathy where it exhibited a strong anti-inflammatory activity marked by the suppressed p38 MAPK and JNK [142]. In models of streptozotocin-induced renal damage in diabetic mice [143], cold-restrained stress and pylorus ligation-induced ulcer [144], ethanol-induced gastric lesion [145], and LPS/D-galactosamine-induced acute liver injury [146], esculin significantly ameliorated inflammation evidenced by the suppressed TNF-*α*, IL-1*β*, and MPO. Li et al. reported the protective role of esculin against LPS-induced macrophages and endotoxin shock in mice and NO production *in vitro*. Esculin inhibited the LPS-induced increase in TNF-*α* and IL-6 and upregulated IL-10 via suppression of NF-*κ*B [147]. Another study by Li et al. concluded that esculin markedly inhibited iNOS/NO levels, and NF-*κ*B protein expression in gastric injury induced by alcohol [145]. Additionally, pretreatment with esculin suppressed TNF-*α* and IL-6 expression [145].

Pertaining to the impact of esculin on Nrf2 activation, Liu et al. have shown that esculin attenuated acute liver injury in mice induced by LPS/D-galactosamine and reduced pathological symptoms of acute hepatic injury via suppression of NF-*κ*B expression as well as activation of Nrf2/HO-1 signaling [146]. Another study by Kim et al. reported that esculin activated Nrf2/ARE signaling in macrophages [148]. Additionally, esculin markedly inhibited neutrophilic lung inflammation which was not recapitulated in Nrf2 knockout mice, suggesting that the anti-inflammatory activity of esculin mainly acts via Nrf2 activation [148].

### 3.8. Esculetin

Esculetin (6,7-dihydroxychromen-2-one) is one of the main bioactive ingredients of *Cortex Fraxini*. Esculetin exhibits a potent antioxidant effect and showed a scavenging activity against DPPH radicals in a time- and concentration-dependent manner [149]. Esculetin has been widely used in antitussive aspects [150], and the study of Liang et al. has demonstrated its protective effect against oxidative stress-induced DNA damage [151]. In addition, esculetin possesses anti-inflammatory [152, 153], antibacterial [154, 155], and antitumor activities against different cancer cells *in vitro* [156–158] and *in vivo* [159, 160]. It has also been reported to enhance the inhibitory effect of 5-fluorouracil on the proliferation of colorectal cancer [161]. The anti-inflammatory activity of esculetin was effective in the inhibition of cartilage destruction in rheumatoid arthritis and osteoarthritis where it suppressed MMP-1 expression in cartilage and decreased NO and PGE2 levels in the synovium [162]. In the context of obesity, esculetin attenuated chronic inflammation by suppressing proinflammatory cytokine release during the interaction between adipocytes and macrophages [163]. In psoriatic mouse skin, esculetin attenuated the disease progression and dramatically decreased proinflammatory cytokines, including TNF-*α*, IL-6, IL-22, IL-23, IL-17A, and IFN-*γ* [152].

Several studies have supported the involvement of Nrf2 signaling in the pharmacological activities of esculetin. For instance, the study of Rubio et al. illustrated the different roles of NF-*κ*B and Nrf2 in the antioxidant imbalance produced by esculetin on leukemia cells and concluded that esculetin resulted in a significant increase in the nuclear translocation of Nrf2 [164]. A recent study conducted by Xu et al. showed that esculetin attenuated neurological defects and alleviated cognitive impairments in transient bilateral common carotid artery occlusion in mice via Nrf2 activation and markedly ameliorated mitochondrial fragmentation and stress [165]. Sen et al. demonstrated the role of esculetin in attenuating the progression of diabetic nephropathy via Nrf2 activation and inhibition of HG-induced ROS production [166]. Han et al. reported that esculetin protected against H_2_O_2_-induced ROS accumulation in the myoblasts through the activation of the Nrf2/NQO1 pathway [167]. Treatment of the pancreatic cancer cell lines with esculetin resulted in significant inhibition of cell proliferation, intracellular ROS, and protein levels of NF-*κ*B [168]. Additionally, esculetin increased the Nrf2 and NQO1 gene expression as well as Nrf2 nuclear accumulation and induced mitochondrial-dependent apoptosis [168]. The same study has shown the binding ability of esculetin to directly bind Keap1 as evidenced by molecular docking and *in vitro* assays [168]. Subramaniam and Ellis reported that esculetin protects HepG2 cells against H_2_O_2_-induced injury via activation of the Nrf2/NQO1 pathway [169]. Pretreatment of the HepG2 cells with esculetin preserved cell integrity following exposure to H_2_O_2_ and increased the nuclear accumulation of Nrf2 [169].

### 3.9. Umbelliferone (UMB)

UMB (7-hydroxychromen-2-one) is a coumarin widely spread in plants belonging to the family *Umbelliferae*. The *Umbelliferae* family is inclusive of economically important herbs, such as celery, cumin, fennel, parsley alexanders, angelica, asafoetida, and giant hogweed [9, 170]. UMB possesses a variety of bioactivities, and several investigators have previously reported its *in vivo* antioxidant and anti-inflammatory effects and studied the underlying mechanisms of action in several animal models [30, 171–175]. The anticancer activity of UMB in hepatocellular [176], colon [177], and oral [178] carcinomas has been well-acknowledged.

Several researchers have demonstrated the strong anti-inflammatory activity of UMB. The *in vivo* anti-inflammatory activity of UMB has recently been reported by Wang et al. who showed its protective effect against acute lung injury induced by LPS [179]. Another study by Yin et al. reported that UMB inhibited inflammation in diabetic mice through suppressing NF-*κ*B and TLR-4. In addition, UMB mitigated hepatic oxidative injury via activating the Nrf2-mediated signal pathway [180]. Li et al. reported that UMB significantly attenuated ROS accumulation and cytotoxicity induced by methylglyoxal (MG) through the activation of Nrf2/ARE signaling [181]. Depletion of Nrf2 by siRNA markedly inhibited the protective effect of UMB against MG-induced alterations, suggesting the key role of Nrf2 in mediating UMB's activity [181]. The role of Nrf2 has been supported by the study of Sen et al. showing that UMB attenuated the progression of diabetic nephropathy via Nrf2 activation and inhibition of ROS production induced by HG [166]. Additionally, Mohamed et al. reported that UMB ameliorated CCl_4_-induced hepatotoxicity in rats through induction of the nuclear translocation of Nrf2, thereby ameliorating oxidative stress-related liver injury via enhancement of cellular antioxidant defenses [138]. Furthermore, UMB protected against renal injury induced by methotrexate (MTX) and attenuated oxidative injury via downregulation of Keap1 and upregulation of Nrf2. The results have also shown that UMB inhibited inflammatory responses via downregulation of both NF-*κ*B and p38 MAPK in the kidney of MTX-intoxicated rats [174].

Previous work from our lab has demonstrated the antioxidant and anti-inflammatory activities of UMB in different animal models [30, 34, 44]. UMB administration mitigated cyclophosphamide-induced oxidative damage and inflammatory response through marked elevation of Nrf2, HO-1, and PPAR*γ* expression. UMB attenuated lipid peroxidation, enhanced antioxidants, and suppressed serum proinflammatory mediators and hepatic iNOS and NF-*κ*B expression. The results showed that coactivation of Nrf2 and PPAR*γ* represents the main mechanism underlying the hepatoprotective effect of UMB [44]. In a rat model of CCl_4_-induced hepatic fibrosis, UMB mitigated inflammation, oxidative injury, and collagen deposition. In addition, UMB suppressed NF-*κ*B p65 and TGF-*β*1/Smad3 and upregulated hepatic PPAR*γ* [34]. Moreover, UMB attenuated lipid peroxidation, NO release, and cerebral inflammation and downregulated nNOS and soluble guanylate cyclase expression in the cerebrum of hyperammonemic rats [30].

### 3.10. Fraxetin

Fraxetin (7,8-dihydroxy-6-methoxychromen-2-one) is a simple coumarin compound extracted from the traditional medicinal plant *Fraxinus rhynchophylla*. Fraxetin is widely available and of relatively low cost and with few side effects. Fraxetin has received recent attention for its antitumor [182–184], radical-scavenging [67, 185–189], anti-inflammatory[190], and antibacterial activities [191]. Fraxetin has also been reported to protect against liver fibrosis induced by CCl_4_ via suppression of the NF-*κ*B signaling pathway as well as phosphorylation of MAPK proteins [192].

The role of Nrf2 in mediating the pharmacologic effects of fraxetin has been demonstrated in few studies. In this context, the protective effect of fraxetin on oxidative stress induced by *Plasmodium berghei* infection in mice has been investigated by Singh et al. [193]. Postinfection treatment of the mice with fraxetin suppressed lipid peroxidation and boosted GSH and antioxidant enzymes. The authors have also reported a significant increase in the serum Nrf2-antioxidant response element level [193]. The effect of fraxetin on HO-1 expression in HaCaT human keratinocytes has been investigated by Kundu et al. [194]. Fraxetin activated the Nrf2/HO-1 pathway in HaCaT cells, induced the nuclear translocation of Nrf2, and increased the ARE-reporter gene activity [194]. Thuong et al. reported that fraxetin inhibited vascular proliferation and atherosclerosis and upregulated HO-1 in vascular smooth muscle cells (VSMCs) [195]. Subcellular fractionation and reporter gene analysis using an ARE construct demonstrated that fraxetin upregulated Nrf2 and reporter activity and concluded that fraxetin has direct protective properties against LDL oxidation via Nrf2/ARE activation [195].

### 3.11. Fraxin

Fraxin (7-hydroxy-6-methoxy-8-[(2*S*,3*R*,4*S*,5*S*,6*R*)-3,4,5-trihydroxy-6-(hydroxymethyl)oxan-2-yl]oxychromen-2-one) is the main bioactive component of the Chinese traditional herb *Cortex Fraxini* [196]. This natural coumarin displayed inspiring biological activities, including anti-inflammatory [197], antioxidant [197, 198], and antihyperuricemic activities [199].

The dual antioxidant and anti-inflammatory activity of fraxin has been recently demonstrated in a mouse model of acute respiratory distress syndrome (ARDS) [200]. Fraxin inhibited the production of TNF-*α*, IL-1*β*, IL-6, ROS, and MDA; suppressed NF-*κ*B, MAPK signaling, and MMP9; and increased SOD in the lung of mice with LPS-induced ARDS [200]. Niu et al. reported that fraxin exhibited hepatoprotective effects against CCl_4_-induced liver damage via mitigation of oxidative stress and inflammation [197]. Fraxin alleviated hepatic injury as indicated by the suppressed production of inflammatory mediators and enhancement of the antioxidant defense mechanisms. An *in vitro* study demonstrated that pretreatment of HepG2 with fraxin protected against the deleterious effects of CCl_4_. Fraxin inhibited CCl_4_-induced MAPK, NF-*κ*B, and COX-2 protein expression [197]. In two different studies conducted by Li et al., the protective effect of fraxin against LPS-induced endotoxic shock [201] and acute lung injury in mice [202] has been investigated. In both studies, the ameliorative effect of fraxin was associated with decreased release of proinflammatory mediators, suppressed ROS generation and oxidative stress, and downregulated NF-*κ*B and NLRP3 inflammasome signaling pathways. These findings highlighted the potent suppressive effect of fraxin on both inflammatory and oxidative responses [201, 202]. In an animal model of I/R-induced kidney injury, fraxin exhibited an ameliorative effect mediated through suppressing oxidative DNA damage and NF-*κ*B [203]. Furthermore, Chang et al. have pointed to the role of Nrf2 in mediating the hepatoprotective efficacy of fraxin [198]. In their study, fraxin markedly inhibited the t-BHP-induced cytotoxicity and ROS generation in HepG2 through Nrf2 pathway-dependent HO-1 expression. *In vivo* studies showed that fraxin has potent hepatoprotective effects against CCl_4_-induced hepatotoxicity in rats via direct antioxidant activity and the Nrf2/ARE pathway [198].

### 3.12. Anomalin

Anomalin ([(9*R*,10*R*)-8,8-dimethyl-9-[(*Z*)-2-methylbut-2-enoyl]oxy-2-oxo-9,10-dihydropyrano[2,3-f]chromen-10-yl] (*Z*)-2-methylbut-2-enoate) is a pyranocoumarin constituent isolated from *Saposhnikovia divaricata*. Anomalin displayed numerous pharmacological effects including anti-inflammatory, antioxidant, and antitumor properties [204–206]. Anomalin protected against acute lung injury induced by LPS via inhibiting the production of TNF-*α*, IL-1*β*, IL-6, and NO [207]. Similarly, anomalin exerted a potent antioxidant activity through increasing enzymatic activities of GST and catalase. Additionally, an *in vitro* study reported that anomalin significantly downregulated the MAPK (p38, JNK, and ERK1/2) in the RAW264.7 cells [207]. In LPS-stimulated macrophages *in vitro*, anomalin inhibited inflammation and NF-*κ*B DNA binding [206].

Anomalin has also exerted a neuroprotective effect in diabetic mice and sodium-nitroprusside- (SNP-) induced neuro-2a cells. Anomalin suppressed neuropathic pain in diabetic mice; abolished iNOS, COX-2, and NF-*κ*B and MAPK signaling in SNP-stimulated cells; and inhibited proinflammatory cytokines in HG-induced primary neurons [208]. The modulatory effect of anomalin on Nrf2 signaling has been scarcely studied. In the study of Khan et al., anomalin reduced Nrf2 and HO-1 gene expression levels which were increased following stimulation of the neuro-2a cells with SNP [208]. However, the exact effect of anomalin on Nrf2 signaling needs to be investigated.

### 3.13. Wedelolactone

Wedelolactone (1,8,9-trihydroxy-3-methoxy-[1]benzofuro[3,2-c]chromen-6-one) is a natural coumarin isolated from *Eclipta prostrata* L. It exhibited an anticancer effect against different tumor cells, such as prostate [209], breast [210], and pituitary adenomas [211]. It has been reported to exert immunomodulatory, anti-inflammatory [212, 213], antimyotoxic, antihemorrhagic [214], and antioxidant activities [215], inhibit osteoclastogenesis, and enhance osteoblastogenesis [216, 217]. Wedelolactone can suppress LPS-induced inflammation in mouse embryo fibroblasts via suppression of NF-*κ*B activity [218].

Wedelolactone protected against quinolinic acid-induced neurotoxicity and impaired motor function through marked inhibition of neuronal TNF-*α*, IL-6, and IL-*β* expression by suppressing NF-*κ*B [212]. Cuong et al. reported that wedelolactone prevented zymosan-induced inflammatory responses in murine bone marrow-derived macrophages through downregulation of TNF-*α* and IL-6 [213]. Zhu et al. have shown that wedelolactone mitigated inflammation and oxidative injury induced by doxorubicin by suppressing the I*κ*K/I*κ*B/NF-*κ*B signaling pathway [215].

Studies showing the modulatory effect of wedelolactone on Nrf2 signaling are very few. Lin et al. reported that wedelolactone inhibited t-BHP-induced damage in PC12 cells and D-galactose-induced neuronal cell loss in mice through improvement of the antioxidant defense capacity via Nrf2/ARE pathway activation [219]. On the other hand, Ding et al. demonstrated that wedelolactone protected human bronchial epithelial cells against cigarette smoke extract-induced oxidative stress and inflammation responses through Nrf2 inhibition [220].

### 3.14. Glycycoumarin

Glycycoumarin (3-(2,4-dihydroxyphenyl)-7-hydroxy-5-methoxy-6-(3-methylbut-2-enyl)chromen-2-one) is a major bioactive coumarin compound isolated from licorice. Given its favorable bioavailability features, glycycoumarin exhibited various pharmacological properties including antioxidant [221, 222], anti-inflammatory [223], and antimicrobial [222, 224] activities. Glycycoumarin dose-dependently inhibited LPS-induced ROS generation in macrophages and effectively suppressed NO, IL-6, and PGE2 expression [223]. Song et al. demonstrated that glycycoumarin attenuated hepatotoxicity induced by alcohol following either chronic or acute ethanol exposure via activation of Nrf2. p62 upregulation by a transcriptional mechanism has also been reported to contribute to Nrf2 activation via a positive feedback loop [225]. In contrast, Yan et al. have demonstrated that Nrf2 was not implicated in the protective effect of glycycoumarin on acetaminophen hepatotoxicity [226].

### 3.15. Osthole

Osthole (7-methoxy-8-(3-methylbut-2-enyl)chromen-2-one) is a coumarin found in a high content in the mature fruit of *Cnidium monnieri* which is commonly applied in the clinical practice of Chinese medicine. Osthole is also widely distributed in other medicinal plants of the genera *Citrus*, *Clausena Angelica*, and *Archangelica*. *Fructus Cnidii* improved male function and reinforced the immune system mainly due to its rich content of osthole [227, 228]. Osthole exhibited various pharmacological activities, including antioxidant [229–231], anticancer [232, 233], and anti-inflammatory properties [231, 234, 235].

Several reports of underlying molecular mechanisms reported that osthole displays a strong anti-inflammatory activity. Fan et al. reported that osthole effectively and safely protected against ulcerative colitis (UC) via marked inhibition of TNF-*α* expression in the colon and markedly reduced MPO activity via suppression of NF-*κ*B p65 and p-I*κ*B*α* [234]. In an *in vitro* study, osthole inhibited the production of TNF-*α*, NO, PGE2, and IL-6 in LPS-induced macrophages [234]. Osthole has a potent selective inhibitory effect on 5-lipoxygenase and COX-1 [236, 237]. It suppressed the immune response of LPS-stimulated macrophages by abolishing ROS generation, iNOS, MAPK, and COX-2 [238, 239]. Osthole also suppressed IL-4- and TNF-*α*-induced eotaxin expression in bronchial epithelial cells [240] and protected against carrageenan-induced hind paw edema in rats via suppression of PG and NO production [241]. Osthole protects against lumbar disc herniation-induced sciatica and relieved mechanical allodynia through decreasing the COX-2 and iNOS expression in the dorsal root ganglion in rats [242].

The modulatory effect of osthole on Nrf2 signaling has been well-acknowledged by different researchers. A recent study conducted by Chu et al. reported that the protective effect of osthole against glutamate-induced Alzheimer's disease in mice was mediated via Nrf2 activation and its downstream antioxidant proteins SOD-1 and HO-1 [243]. Osthole protected against Ang II-induced apoptosis of rat aortic endothelial cells through suppression of NF-*κ*B and activation of Nrf2 and its downstream antioxidant genes and effectively inhibited Keap1, denoting its potential therapeutic effect against vascular injury [244]. In another study, osthole protected against LPS-induced inflammation in BV2 cells via NF-*κ*B suppression and upregulation of the Nrf2/HO-1 pathway dose dependently [245]. Additionally, osthole exerted neuroprotective effects against global cerebral I/R injury by reducing oxidative stress via the upregulation of the Nrf2/HO-1 signaling pathway [246]. The renoprotective effect of osthole against accelerated focal segmental glomerulosclerosis was mediated via Nrf2 activation and subsequently downregulation of the NF-*κ*B-mediated COX-2 expression [247]. Osthole has also shown renoprotective effects mediated through inhibition of ROS generation and NF-*κ*B/NLRP3 signaling and increased Nrf2 nuclear translocation [248]. Osthole has protective effects on LPS-induced acute lung injury by upregulating the Nrf-2/Trx-1 pathway, whereas Nrf2 siRNA blocked its beneficial effects [249].

### 3.16. Hydrangenol

Hydrangenol (8-hydroxy-3-(4-hydroxyphenyl)-3,4-dihydroisochromen-1-one) is a natural dihydroisocoumarin mostly obtained from the *Hydrangea* species (*Hydrangeaceae*) leaves. Hydrangenol possesses anti-inflammatory [250], antidiabetic [251], antioxidant [250, 252], anticancer [253], and antiangiogenic activities [254]. It inhibited LPS-induced NO release and iNOS expression via suppression of NF-*κ*B and consequently inhibiting NF-*κ*B-DNA. Additionally, hydrangenol suppresses NO production by inducing HO-1 and promoting nuclear translocation of Nrf2. In contrast, transient knockdown of Nrf2 markedly inhibited hydrangenol-induced HO-1 expression, indicating that hydrangenol-induced Nrf2 is an upstream regulator of HO-1 [252]. Hydrangenol exerted antiphotoaging activity *in vitro* and in UVB-irradiated HR-1 hairless mice. Hydrangenol effectively reduced MMP-1/-3, COX-2, and IL-6 expression and attenuated the phosphorylation of MAPKs and STAT1. Interestingly, hydrangenol upregulated the expression of Nrf2, HO-1, NQO1, GCLM, and GCLC [252].

### 3.17. Isoimperatorin

Isoimperatorin (4-(3-methylbut-2-enoxy)furo[3,2-g]chromen-7-one) is a 6,7-furanocoumarin derivative. This compound is present in *Angelica dahurica*, *Notopterygium incisum*, *Ferula lutea*, *Angelica pubescens*, and *Peucedanum praeruptorum*. Isoimperatorin exhibits various pharmacological activities, including antioxidant [255, 256], anti-inflammatory [257], analgesic [258], antibacterial [259, 260], and anticancer properties [261, 262].

Wijerathne et al. reported that isoimperatorin protected against OA-induced asthma via mitigation of airway inflammation and mucus hypersecretion evidenced by the decreased IL-4, IL-5, and IL-13 production [257]. Mechanistically, isoimperatorin suppressed the activation of NF-*κ*B, p38 MAPK, and ERK1/2 [257]. In addition, isoimperatorin inhibited TNF-*α*-induced vascular cell adhesion molecule-1 and ROS production and upregulated the PPAR*γ* signaling pathway in human endothelial cells [263]. It has a strong hepatoprotective effect against cytotoxicity in H4IIE cells induced by aflatoxin B1 via Nrf2/ARE activation and induction of GST-*α* and suppression of CYP1A expression [256].

The effects of coumarins on Nrf2 signaling in *in vivo* and *in vitro* studies are summarized in Tables 1 and 2, respectively.

## 4. *In Silico* Evidence for Binding of Coumarins to Keap1 Protein

**Figure 3:**
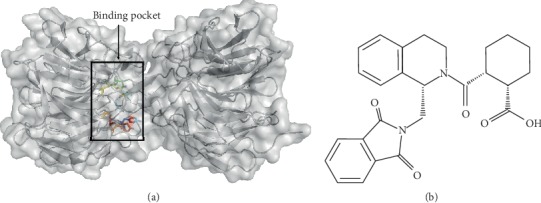
Surface map of Keap1 protein (a) and chemical structure of (*S*,*R*,*S*) (b). The colored residues represent the active site of Keap1 which is involved directly in the interaction with the inhibitor (*S*,*R*,*S*) (PDB ID: 4l7b) and include Y334, S363, R380, N414, R415, S508, S555, Y572, and S602.

**Figure 4:**
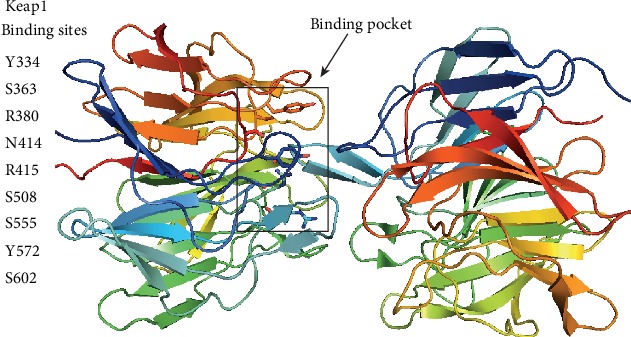
The binding pocket of Keap1 includes Y334, S363, R380, N414, R415, S508, S555, Y572, and S602. These residues were used for the site-specific docking of coumarin derivatives into Keap1.

Keap1 plays the key step in the ubiquitination and degradation of Nrf2. In this review, we aimed to provide *in silico* evidence that coumarins bind Keap1 and hence could be employed as promising Nrf2 activators. AutoDock Vina 1.5.6 was used to perform molecular docking of coumarin derivatives and Keap1 protein. The complex structure of Keap1 with (1*S*,2*R*)-2-[(1*S*)-1-[(1,3-dioxo-2,3-dihydro-1H-isoindol-2-yl)methyl]-1,2,3,4-tetrahydroisoquinolin-2-carbonyl]cyclohexane-1-carboxylic acid (compound (*S*,*R*,*S*)) with PDB ID: 4l7b was used as a model for the docking study (Figure 3). The binding pocket of Keap1 was used to identify the binding conformation of the different coumarin derivatives (Figure 4). The average of the lowest energy of docking was used to show the binding affinity for each coumarin derivative with Keap1. The best-scored conformation has been chosen and visually analyzed using the PyMOL 1.7.6 software. Briefly, 9 different orientations were generated, and the first pose with the lowest docking energy has been used. Each coumarin has a different conformation; however, all the conformations were allocated in the vicinity of the active site. The mean docking energy of all 9 generated models was calculated.

There are very limited biophysical studies that include the experimental binding data of all listed coumarin derivatives and Keap1. Therefore, this review sheds light on the promising compounds which should be studied in the future. To perform an experimental study, the Keap1 protein needs to be expressed and purified, and the binding affinities with different coumarin derivatives could be estimated using isothermal titration calorimetry (ITC) and/or surface plasmon resonance (SPR). Based on the binding affinity, crystallization trials could be conducted to get an accurate binding mode between Keap1 and coumarins. This review highlights the promising target for biophysical studies.

**Figure 5:**
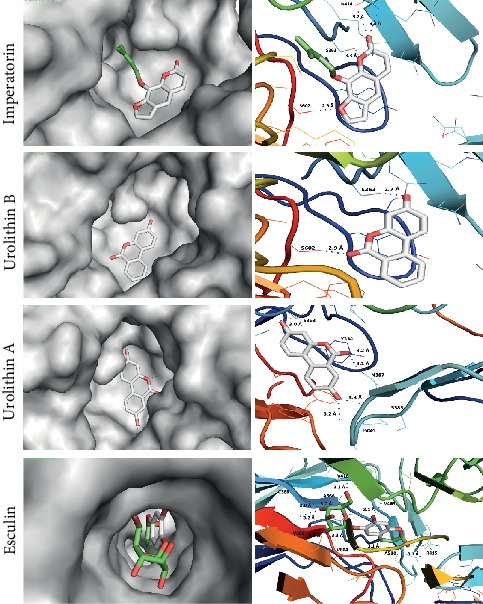
Docking models of imperatorin, urolithin B, urolithin A, and esculin with Keap1. All the compounds are rich with polar groups and form hydrogen bonds with the polar, negatively, and positively charged amino acids in the vicinity of the active site of Keap1.

**Figure 6:**
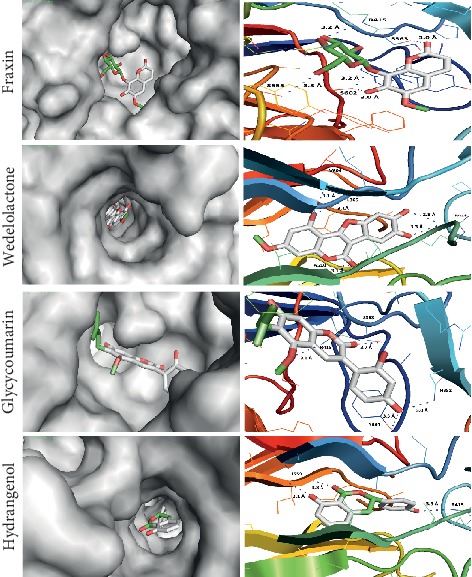
Docking models of fraxin, wedelolactone, glycycoumarin, and hydrangenol with Keap1. All the compounds are rich with polar groups and form hydrogen bonds with the polar, negatively and positively charged amino acids in the vicinity of the active site of Keap1.

We performed docking for (*S*,*R*,*S*) which naturally cocrystallizes and binds Keap1 with high affinity. This is to obtain the lowest energy score of docking as this energy represents the reference to judge the predicted binding affinity of different coumarin derivatives with Keap1. Most of the coumarin derivatives showed promising inhibitory effect on Keap1 based on the lowest energy score of docking. Eight derivatives (IMP, urolithin B, urolithin A, esculin, fraxin, wedelolactone, glycycoumarin, and hydrangenol) showed better binding with Keap1, and their affinities are quite close to the (*S*,*R*,*S*) compound as shown in Table 3. These eight compounds are well fitted in the vicinity of the binding pocket through forming hydrogen bonds with the side chains of the polar, positively, and negatively charged amino acids as illustrated in Figures 5 and 6. These results show that esculin and wedelolactone are the most promising coumarins for the development of pharmacological Keap1 inhibitors/Nrf2 activators.

*IMP*: the carbonyl group of a coumarin moiety forms a hydrogen bond and n⟶*π*∗ interaction with the amino group and carbonyl group of NH_2_ of the side chain of N414, respectively. Also, the oxygen atom of furan and pyran rings forms two hydrogen bonds with the -OH group of the side chain of S602 and S363, respectively.

*Visnagin*: the carbonyl group and the oxygen of the methoxy group of the coumarin moiety form two hydrogen bonds with the -OH group of the side chain of S602, while the oxygen of the furan ring forms a hydrogen bond with the amino group of the side chain of N387 at the dimeric interface of the Keap1 protein.

*Urolithin B*: the carbonyl and hydroxy groups of the coumarin moiety exhibited two hydrogen bonds with S602 and S363, respectively. These two hydrogen bonds have a remarkably short length approximately 2.9 Å and 2.7 Å which could enhance the interaction of urolithin B and binding pocket of Keap1.

*Urolithin A*: this forms five hydrogen bonds at the dimeric interface of the Keap1 protein. Both hydroxyl groups form hydrogen bonds with S363, P384, and S383. Also, the carbonyl groups form two hydrogen bonds with the -OH group of the side chain of Y334 and the amino group of the side chain of N387.

*Daphnetin*: the carbonyl oxygen forms a hydrogen bond with S602, and the two hydroxy groups form two hydrogen bonds with S363.

*Esculin*: this coumarin is rich in hydroxyl groups which serve as hydrogen bond donors to the residues in the active site of Keap1. It forms six hydrogen bonds with the carbonyl group of C368, A366, V418, V604, and V606. These hydroxyl groups of the oxan ring support the perfect fitting of esculin deep in the binding pocket of Keap1. In addition, the hydroxyl group of the coumarin moiety forms two hydrogen bonds with V463 and A510. The carbonyl group of the lactone ring forms a hydrogen bond with the amino group of the side chain of R415. Esculin makes hydrophobic interactions with the nonpolar amino acids in V418, V604, V606, V463, and A510.

*UMB*: this forms two hydrogen bonds with S363 and N387 at the dimeric interface of Keap1.

*Fraxetin*: the dihydroxy groups form bonds with the side chain of N382 and S363, and the oxygen of the methoxy group forms a hydrogen bond with S363. The carbonyl group and oxygen of the pyran ring form two extra hydrogen bonds with N387. These five hydrogen bonds are located at the dimeric interface of Keap1.

*Fraxin*: the hydroxyl groups of oxan rings and oxygen of the ether bond between the oxan ring and the coumarin nucleus form three hydrogen bonds with the side chain of R415, S555, and S602. Also, the hydroxyl and carbonyl groups of this coumarin form two hydrogen bonds with S602 and S363, respectively. The coumarin nucleus exhibits a different conformation in fraxin as compared to esculin which could prevent the perfect fit of fraxin in the binding pocket of Keap1. This explanation is supported by the lower energy of the docking of esculin than fraxin.

*Esculetin*: this forms six hydrogen bonds with S602, N382, N387, and Y334. Also, the benzene ring of the coumarin nucleus makes hydrophobic interaction with the aromatic ring of Y334.

*Anomalin*: this forms two hydrogen bonds with R415 and S602.

*Wedelolactone*: this fits properly in the active site of Keap1 due to its unique conformation. The hydroxyl group of the coumarin moiety forms two hydrogen bonds with the carbonyl groups of L365 and V604. The carbonyl group of coumarin exhibits n⟶*π*∗ interaction with the carbonyl group of A510. The two-hydroxyl group of the benzofuran ring forms two hydrogen bonds with the side chains of R415. Wedelolactone has hydrophobic interactions with L365, A366, V604, and A510.

*Glycycoumarin*: the oxygens of the methoxy and carbonyl groups of coumarin form two hydrogen bonds with R415 and S363. Another two hydrogen bonds are formed between the hydroxy group of the dihydroxyphenyl group and the side chain of N382 and Y334. The phenyl group of glycycoumarin has a hydrophobic interaction with the aromatic ring of the side chain of Y334.

*Scopoletin*: this forms six hydrogen bonds with the side chains of S363, N414, N382, and Y334 at the dimeric interface of Keap1. Also, there is n⟶*π*∗ interaction between the carbonyl group of coumarin and the carbonyl group of R336.

*Osthole*: this forms three hydrogen bonds with the side chains of N387 and S602 at the dimeric interface of Keap1.

*Hydrangenol*: this forms three hydrogen bonds with I559 and the side chain of R415. It has a hydrophobic interaction with A556, V604, I559, and L577.

*Isoimperatorin*: this forms two hydrogen bonds with the side chains of S602 and N387 at the dimeric interface of Keap1.

## 5. Conclusions

The Nrf2 signaling pathway plays a vital role in the protection against cell injury induced by oxidative stress and electrophiles. Excessive production of ROS and oxidative injury are associated with the pathogenesis of several diseases and disorders. There is crosstalk between the Nrf2/ARE signaling and other signaling pathways, including NF-*κ*B, and this represents the main underlying mechanism by which Nrf2 exerts its anti-inflammatory activities. Given the dual role of Nrf2 activation in the prevention of oxidative stress and inflammation, pharmacological activation of this signaling pathway could represent a powerful strategy for treating diseases associated with excessive release of ROS and proinflammatory mediators. Coumarins represent a vast group of natural compounds with beneficial pharmacological properties. Several studies have demonstrated the role of Nrf2 activation in mediating the therapeutic effects of some coumarins both *in vitro* and *in vivo*. We employed molecular docking simulations to investigate the binding of coumarins with Keap1 and to verify whether their therapeutic effects are related to antioxidant properties mediated via Nrf2 modulation. IMP, visnagin, urolithin B, urolithin A, scopoletin, esculin, esculetin, UMB, fraxetin, fraxin, daphnetin, anomalin, wedelolactone, glycycoumarin, osthole, hydrangenol, and isoimperatorin showed binding affinities toward Keap1 through forming hydrogen bonds with the side chains of the polar, positively, and negatively charged amino acids. IMP, urolithin B, urolithin A, esculin, fraxin, wedelolactone, glycycoumarin, and hydrangenol exhibited strong binding with Keap1 with affinities quite close to a standard Keap1 inhibitor. Thus, activation of the Nrf2 signaling pathway can explain some of the pharmacological and biological effects of coumarins. Coumarins therefore represent promising leads in the development of effective Keap1 inhibitors/Nrf2 activators and a good resource for discovering drug candidates for the treatment/prevention of various diseases.

## Figures and Tables

**Table 1:** Effect of coumarins on Nrf2 in animal models of different diseases.

Coumarin	Disease/model	Animal	Effects	Reference(s)
IMP	Nrf2 knockout	Mice	IMP induced hepatic antioxidant activities via the Nrf2/ARE mechanism. IMP induced hepatic GST and/or NQO1 activities.	[75]
	Allergic responses mediated by mast cells	Mice	IMP attenuated allergic responses. IMP inhibited mast cell degranulation, MAPK, NF-*κ*B, and inflammatory mediators' expression. IMP activated PI3K/Akt and Nrf2/HO-1 pathways.	[77]

Visnagin	Cerulein-induced acute pancreatitis	Mice	Visnagin upregulated Nrf2 and attenuated oxidative stress. Visnagin mitigated pancreatic inflammation and NF-*κ*B p65 nuclear translocation.	[86]

Urolithin B	LPS-induced systemic inflammation	Mice	Urolithin B reduced intracellular ROS production and NADPH oxidase expression. Urolithin B upregulated AMPK phosphorylation and Nrf2/ARE signaling and HO-1 expression.	[92]

Urolithin A	Colitis	Mice	Urolithin A enhanced gut barrier function and inhibited inflammation through Nrf2-dependent pathways.	[104]
	High cholesterol diet-fed rats	Rats	Urolithin A upregulated aortic scavenger receptor-class B type I expression and Nrf2 and inhibited ERK1/2 phosphorylation levels.	[264]

Scopoletin	MG-induced hyperglycemia and insulin resistance	Rats	Scopoletin increased insulin sensitivity, decreased AGEs, and activated Nrf2 by Ser40 phosphorylation.	[121]

Daphnetin	Cisplatin-induced nephrotoxicity	Mice	Daphnetin inhibited ROS generation, lipid peroxidation, NF-*κ*B activation, and proinflammatory cytokines. Daphnetin upregulated Nrf2 and HO-1 expression.	[132]
	CCl_4_-induced hepatotoxicity	Rats	Daphnetin improved liver function, inhibited histological alterations and lipid peroxidation, and increased Nrf2 and HO-1 gene expression.	[138]
	7,12-Dimethylbenz[a]anthracene-induced mammary carcinogenesis	Rats	Daphnetin inhibited lipid peroxidation, enhanced GSH and antioxidant enzymes, decreased NF-*κ*B expression, and activated Nrf2 pathway.	[265]

Esculin	LPS/D-galactosamine-induced liver injury	Mice	Esculin suppressed lipid peroxidation, MPO, TNF-*α*, IL-1*β*, and NF-*κ*B and increased the expression of Nrf2 and HO-1.	[146]

Esculetin	Cerebral I/R	Mice	Esculetin ameliorated mitochondrial oxidative stress, fragmentation, and stress and increased SOD and Nrf2 expression.	[165]

UMB	Hepatic injury in diabetic db/db mice	Mice	UMB ameliorated liver function, serum lipids, and lipid peroxidation and suppressed NF-*κ*B and TLR-4. UMB activated Nrf2 signaling pathway.	[180]
	Cyclophosphamide-induced hepatotoxicity	Rats	UMB ameliorated liver function and inhibited histological alterations, lipid peroxidation, and inflammation. UMB upregulated Nrf2, HO-1, PPAR*γ*, and antioxidants and suppressed iNOS and NF-*κ*B.	[44]
	CCl_4_-induced hepatotoxicity	Rats	UMB improved liver function, inhibited histological alterations and lipid peroxidation, and increased Nrf2 and HO-1 gene expression.	[138]
	MTX-induced nephrotoxicity	Rats	UMB inhibited inflammatory response via downregulation of both NF-*κ*B and p38 MAPK genes. UMB downregulated Keap1 and upregulated Nrf2.	[174]

Fraxetin	Malaria infection	Mice	Fraxetin suppressed lipid peroxidation and boosted GSH and antioxidant enzymes via Nrf2-ARE activation.	[193]

Fraxin	CCl_4_-induced hepatotoxicity	Rats	Fraxin ameliorated liver function and lipid peroxidation and increased GSH and Nrf2-mediated antioxidant enzyme system.	[198]

Glycycoumarin	Acute alcoholic liver injury	Mice	Glycycoumarin prevented liver injury via induction of autophagy and activation of Nrf2 signaling.	[225]

Osthole	Alzheimer's disease model	Mice	Osthole restored the mitochondrial membrane potential, ameliorated apoptosis markers, and activated Nrf2 and its downstream antioxidant proteins.	[243]
	Transient global brain ischemia	Mice	Osthole improved the cognitive functions and upregulated Nrf2/HO-1 signaling pathway.	[246]
	Focal segmental glomerulosclerosis	Mice	Osthole suppressed NF-*κ*B-mediated COX-2 expression, PGE2 production, apoptosis, and podocyte injury and activated Nrf2.	[247]
	IgA nephropathy	Mice	Osthole inhibited excessive ROS generation and NF-*κ*B/NLRP3 signaling and increased Nrf2 nuclear translocation.	[248]
	LPS-induced acute lung injury	Mice	Osthole upregulated Nrf-2/thioredoxin 1 and prevented lung injury.	[249]

Hydrangenol	UVB-irradiated hairless mice	Mice	Hydrangenol downregulated MMP-1/-3, COX-2, IL-6, MAPKs, and STAT1 and upregulated Nrf2, HO-1, NQO1, GCLM, and GCLC.	[252]

**Table 2:** Effect of coumarins on Nrf2 in *in vitro* studies.

Coumarin	Model/cells	Effects	Reference(s)
IMP	Arsenic trioxide-induced toxicity in H9c2 cells	IMP-attenuated ROS generation, cytotoxicity, and apoptosis triggered Nrf2 activation.	[76]
	IgE-mediated allergic responses in RBL-2H3 cells	IMP inhibited mast cell degranulation; suppressed NF-*κ*B, p38, JNK, and ERK MAPKs; and increased Nrf2 nuclear translocation.	[77]

Urolithin B	LPS-induced BV2 microglial cells	Urolithin B reduced ROS production, NADPH oxidase expression, NF-*κ*B, ERK, and JNK and increased AMPK phosphorylation, Nrf2, and HO-1.	[92]

Urolithin A	Senescent human skin fibroblasts	Urolithin A increased type I collagen expression, reduced intracellular ROS, abolished MMP-1 expression, and activated Nrf2/ARE signaling.	[103]
	LPS-induced Caco2 and HT-29 cells	Urolithin A activated aryl hydrocarbon receptor- (AhR-) Nrf2-dependent pathways.	[104]

Scopoletin	Rotenone-stimulated SH-SY5Y cells	Scopoletin prevented oxidative stress and apoptosis and activated Nrf2 signaling.	[122]

Daphnetin	Oleic acid-induced HepG2 cells	Daphnetin decreased CYP2E1 and CYP4A expression, promoted glucose uptake and insulin sensitivity, and enhanced PI3K/Akt and Nrf2 signaling.	[133]
	Arsenic-induced human lung epithelial cells	Daphnetin reduced ROS, JNK, ERK, Keap1, and apoptosis and activated Nrf2/ARE pathway.	[134]
	HG-induced human glomerular mesangial cells	Daphnetin reduced ROS production; attenuated the release of IL-1*β*, IL-6, and TNF-*α* via suppression of NF-*κ*B pathway; inhibited the expression Keap1; and upregulated Nrf2.	[135]
	Oxygen-glucose deprivation/reoxygenation-induced hippocampal neurons	Daphnetin inhibited oxidative stress and cell apoptosis and enhanced the nuclear translocation of Nrf2 and HO-1 expression.	[136]
	t-BHP-induced RAW264.7 cells	Daphnetin suppressed ROS, inhibited cytochrome c release and NLRP3 inflammasome activation, and upregulated Nrf2 nuclear translocation along with Keap1 protein downregulation.	[137]

Esculin	EK 293 and RAW264.7 cells	Esculin suppressed ROS production and activated Nrf2/ARE signaling.	[148]

Esculetin	NB4 leukemia cells	Esculetin increased the nuclear translocation of Nrf2.	[164]
	HG-induced rat mesangial cell line HBZY-1	Esculetin suppressed ROS production and IL-6 expression and activated Nrf2.	[166]
	H_2_O_2_-induced C2C12 myoblasts	Esculetin suppressed ROS production and activated the Nrf2/NQO1 pathway.	[167]
	Pancreatic carcinoma cells (PANC-1)	Esculetin increased Nrf2 and NQO1 gene expression and Nrf2 nuclear accumulation.	[168]

	H_2_O_2_-induced HepG2 cells	Esculetin activated Nrf2/NQO1 pathway	[169]
UMB	HG-induced mesangial cells	UMB suppressed ROS production and activated Nrf2.	[166]
	MG-induced HepG2 cells	UMB abolished ROS generation and increased Nrf2 expression, effects inhibited by Nrf2 depletion.	[181]

Fraxetin	Vascular smooth muscle cells	Fraxetin increased the expression of HO-1 and Nrf2.	[195]
	HaCaT human keratinocytes	Fraxetin reduced ROS and upregulated Akt, AMPK, HO-1, and Nrf2.	[194]

Fraxin	t-BHP-induced HepG2 cells	Fraxin inhibited t-BHP-induced cytotoxicity and ROS generation through Nrf2-dependent HO-1 expression.	[198]

Wedelolactone	t-BHP-induced adrenal pheochromocytoma cells	A wedelolactone-rich extract prevented apoptosis and activated Nrf2/ARE pathway.	[219]

Osthole	LPS-stimulated BV2 mouse microglia	Osthole suppressed NF-*κ*B, IL-1*β*, IL-6, and TNF-*α* and upregulated Nrf2/HO-1 signaling	[245]
	LPS-stimulated mesangial cells	Osthole inhibited ROS generation, MCP-1 secretion, and NF-*κ*B activation and upregulated Nrf2.	[248]

Hydrangenol	LPS-stimulated BV2 microglial cells	Hydrangenol attenuated NO production and iNOS expression by inhibiting NF-*κ*B activation and stimulated Nrf2/HO-1 signaling pathway.	[250]

Isoimperatorin	Aflatoxin B1-inducible cytotoxicity in H4IIE	Isoimperatorin activated Nrf2/ARE and GST-*α* and suppressed CYP1A expression.	[256]

**Table 3:** Molecular docking of coumarin derivatives as potential inhibitors of Keap1.

Compounds	Lowest energy of docking (kcal/mol)
IMP	−8.078 ± 0.28
Visnagin	−7.33 ± 0.44
Urolithin B	−8.02 ± 0.43
Urolithin A	−8.01 ± 0.62
Scopoletin	−6.72 ± 0.28
Daphnetin	−6.50 ± 0.20
Esculin	−9.31 ± 0.31
Esculetin	−6.80 ± 0.18
UMB	−6.51 ± 0.15
Fraxetin	−7.02 ± 0.30
Fraxin	−8.20 ± 0.47
Anomalin	−7.21 ± 0.70
Wedelolactone	−9.30 ± 0.33
Glycycoumarin	−8.62 ± 0.53
Osthole	−7.50 ± 0.38
Hydrangenol	−8.41 ± 0.21
Isoimperatorin	−7.60 ± 0.42
Standard (*S*,*R*,*S*)	−10.71 ± 0.40

(*S*,*R*,*S*) is a synthetic compound that crystalized with Keap1 with high affinity (PDB ID: 4l7b) and is used as a standard control for comparison.
